# Relationship Between Neurologic Symptoms and Signs and FMR1 Genotype in Premutation Carriers

**DOI:** 10.1002/acn3.70375

**Published:** 2026-03-31

**Authors:** Flora Tassone, Freddy Chafota, Miguel E. Rentería, Randi J. Hagerman, Ellery Santos, Anna Atkinson, Nicholas G. Martin, Elsdon Storey, Danuta Z. Loesch

**Affiliations:** ^1^ Department of Biochemistry and Molecular Medicine University of California, Davis California USA; ^2^ School of Medicine and MIND Institute University of California Davis Medical Center, Davis California USA; ^3^ Brain and Mental Health Program QIMR Berghofer Medical Research Institute Brisbane Australia; ^4^ School of Biomedical Sciences, Faculty of Health, Medicine and Behavioural Sciences The University of Queensland Brisbane, Queensland Australia; ^5^ Department of Pediatrics University of California Davis Health Sacramento California USA; ^6^ Medical Investigation of Neurodevelopmental Disorders (MIND) Institute University of California Davis Health Sacramento California USA; ^7^ School of Psychology and Public Health La Trobe University Bundoora Victoria Australia; ^8^ Department of Medicine (Neuroscience) Monash University Melbourne Australia

**Keywords:** CGG expansion, *FMR1* premutation carriers, FXTAS, mRNA predictors, neurological involvement

## Abstract

**Background and Objectives:**

Fragile X‐associated Tremor/Ataxia Syndrome (FXTAS) is the most severe late‐onset condition caused by a premutation in the *FMR1* gene, characterized by expanded CGG triplet repeats of 55–200. Clinical presentations of FXTAS, including gait ataxia, kinetic tremor, cognitive decline, and rare Parkinsonism, are linked to white matter degeneration, predominantly in the middle cerebellar peduncles. Underlying pathophysiological mechanisms involve the sequestration of CGG‐binding proteins due to elevated *FMR1* mRNA and repeat‐associated non‐AUG (RAN)‐initiated translation. Outside of the full presentation of FXTAS, some FMR1 premutation carriers exhibit only isolated clinical changes occurring in this syndrome. This study explored the relationship of molecular predictors of disease, either with these isolated features in patients who did not meet diagnostic criteria for FXTAS or with diagnosable FXTAS.

**Methods:**

176 male (*N* = 111) and female (*N* = 65) premutation carriers were separated into three groups based on neurological/cognitive examination data: asymptomatic, non‐syndromic/presenting isolated changes, and syndromic‐FXTAS. These categories were then separately correlated with CGG repeat length and FMR1 mRNA expression levels.

**Results:**

Regression and distributions' analyses showed that the most consistent associations of both genomic markers were with neurological severity rankings, followed by a binary definition of FXTAS status. Among other minor presentations, Parkinsonism and cognitive impairment were significantly correlated with CGG size in male samples.

**Discussion:**

This data provides evidence for a linear relationship between *FMR1* CGG size and mRNA levels, as well as both syndromic and non‐syndromic forms of neurological manifestations, which represent aspects of the premutation‐linked neurodegenerative process that persist with advancing age.

## 1 Introduction

The *FMR1* X‐linked gene premutation, characterized by CGG repeat expansions ranging from 55 to 200, manifests through a diverse spectrum of abnormal conditions [1, 2], including the Fragile X‐associated Tremor/Ataxia Syndrome (FXTAS) [3], a severe late‐onset neurodegenerative condition characterized by progressive gait ataxia, kinetic tremor, cognitive decline, and, less frequently, Parkinsonian features. Its age‐dependent penetrance affects up to 75% of male carriers and approximately 16% of female carriers [4].

The clinical presentation typically correlates with white matter degeneration, predominantly affecting the middle cerebellar peduncles, and the widespread distribution of intranuclear inclusions within neurons and astroglia [5]. The underlying pathophysiological mechanisms of these neurodegenerative alterations are primarily attributed to *FMR1* messenger RNA (mRNA) toxicity, with expression levels elevated 2–10‐fold above normal parameters in a CGG repeat length‐dependent manner [6]. The prevailing RNA gain‐of‐function model postulates pathogenic effects through sequestration of specific RNA‐binding proteins, resulting in subsequent cellular dysfunction [7]. Disruption of the local axonal translation machinery leads to the accumulation of toxic mRNA species within dendritic and axonal compartments. Additionally, pathological processes involve repeat‐associated non‐AUG (RAN)‐initiated translation, generating intracellular deposits of toxic FMRpolyG aggregates and triggering downstream DNA damage responses [2, 8].

Most molecular studies, including those examining genotype–phenotype relationships, have been conducted in carriers affected with FXTAS who meet the established clinical criteria [9]. However, demonstration of the presence of intranuclear inclusions typical of FXTAS in both affected and unaffected premutation females provided evidence for an accumulation of subclinical pathological processes beyond the diagnosable syndrome [6]. This finding helped explain the occurrence of less severe manifestations of FXTAS [10], non‐syndromic (monosymptomatic/subclinical) forms of relevant motor involvement [11, 12] or sole manifestations in emotional domains [2]. The latest presentation of the series of premutation females manifesting psychiatric symptoms, mild cognitive deficits, and subclinical motor symptoms, combined with white matter alterations, reinforced the earlier reports [13].

The associations between various forms of phenotypic manifestations and *FMR1*‐CGG expansion size confirm that premutation alleles are causative of, or contributory to, specific changes observed in these alleles' carriers. Evidence for such relationships has been obtained for the presence or absence, the level of severity, and age of onset of FXTAS, and in rare studies, for the severity of FXTAS‐like single motor impairments [14, 15, 16]. However, these results for cognitive measures or psychiatric outcomes were much less consistent [17, 18].

Here, we address the issue of the dynamics of the pathogenic effect of the *FMR1* genotype on carriers' neurological phenotype by exploring the effect of this genotype on and beyond diagnosable FXTAS. Data from a large sample of adult male and female premutation carriers was applied in correlations between FXTAS status, FXTAS considered as part of the spectrum, including lesser neurological involvement categories, and four individual features, including Parkinsonism, cognitive impairment, depression, and anxiety, with the size of the CGG expansion and expression levels of *FMR1* mRNA. The results showing associations between premutation genotypes and either syndromic or non‐syndromic neurological, motor, and cognitive changes support the hypothesis of a continuity of an underlying pathological process linked to the *FMR1* premutation allele, with age‐ and individual‐dependent, diverse final outcomes.

## 2 Methods

### 2.1 Ethics Statement

This study was conducted in accordance with the principles of the Declaration of Helsinki and was approved by the relevant institutional ethics committees. The Australian components of the study were approved by the La Trobe University Human Research Ethics Committee under the protocols HEC01‐85 and HEC15‐058. Research activities conducted at the University of California, Davis, were approved by the Institutional Review Board in compliance with federal regulations and institutional policies governing human subject research. All study participants provided written informed consent prior to enrolment and participation in any research procedure. The consent process included comprehensive disclosure of the study objectives, procedures, potential risks and benefits, and participants' right to withdraw from the study at any time without penalty. The study protocols adhered to established ethical standards for research involving human subjects, ensuring participant safety, confidentiality, and data protection throughout all phases of the investigation.

### 2.2 Study Participants

This study utilized DNA and clinical data obtained from two independent cohorts of adult carriers of the *FMR1* premutation. The combined sample consisted of 241 participants: 41 Australian carriers recruited through La Trobe University (Melbourne, Australia) and 200 American carriers from the MIND Institute at the University of California, Davis (USA).

The Australian cohort comprised adult premutation carriers, ranging in age from 42 to 80 years (mean = 62.3 years, SD = 8.7). The majority of participants were of European descent, with two exceptions: one male of East Asian (Chinese) origin and one female of Filipino background. The recruitment pathways included referrals from the Victorian Genetic Counseling Clinic at the Murdoch Children's Research Institute, as well as neurology clinics affiliated with the University of Melbourne and Monash University. A minority of the participants, including those residing in other Australian states, were self‐referred through community engagement efforts coordinated by the Australian Fragile X Association.

The American cohort comprised 200 *FMR1* premutation carriers aged 40–85 years (mean = 63.8 years, SD = 9.3). Participants were enrolled in ongoing research studies at the MIND Institute focusing on the clinical and molecular characteristics of premutation carriers. Recruitment primarily occurred through cascade testing of relatives of probands diagnosed with Fragile X Syndrome (FXS). The cohort was predominantly of European ancestry, with approximately one‐third of the patients identified as Hispanic or Latino. Eligibility criteria for inclusion were confirmed *FMR1* premutation carrier status, age above 40 years, and absence of preselection based on the clinical phenotype.

Clinical and molecular data from both cohorts were previously utilized in earlier research exploring the role of genetic modifiers in manifestations of neuropsychiatric and motor features of the FXTAS spectrum [19].

### 2.3 Assessment of Neurological, Cognitive, and Psychiatric Status

In the American cohort, neurological assessments and FXTAS diagnoses were performed by an experienced clinician (RJH), whereas the Australian cohort underwent comprehensive evaluations over a two‐decade period conducted jointly by two experienced neurologists (DZL and ESt). Subsets of both cohorts were monitored longitudinally, and the most recent clinical assessments were used for the present analysis.

FXTAS diagnosis was established according to standardized criteria originally defined in the initial characterization of this syndrome [20] with subsequent modifications documented in later revisions [9]. In the American sample, the diagnostic framework employed a hierarchical classification system: ‘Definite’ FXTAS, representing the most severe phenotype, required the presence of one major clinical feature accompanied by one major radiological characteristic, specifically T2‐weighted hyperintensity within the middle cerebellar peduncles (MCP sign). ‘Probable’ FXTAS required either two major clinical features or one major clinical feature with one minor radiological finding, characterized by white matter changes within the corpus callosum. ‘Possible’ FXTAS encompassed cases of diagnostic uncertainty where only two clinical features (one major and one minor) were present. Female carriers typically manifest within the latter two diagnostic categories (probable or possible) owing to the relative absence of the pathognomonic MCP sign. In the Australian sample, ‘possible FXTAS’ was not included in a diagnosable FXTAS category. Therefore, the unified classification of neurological involvement across the combined cohort used in this study was based on harmonized criteria established between the two study sites.

FXTAS was evaluated using two complementary approaches: first, as a dichotomous variable denoting the presence or absence of a diagnosis, and second, according to a graded scale of neurological involvement that extends beyond syndromic presentations through hierarchical rankings encompassing asymptomatic (unaffected), non‐syndromic (including subclinical subcategory), as well as diagnosable FXTAS categories. Subclinical subcategory represented obvious change(s) disclosed by comprehensive testing but not reported by a participant.

Additional neurological phenotypes examined included Parkinsonism. It was diagnosed based on the manifestation of at least one of the three cardinal motor symptoms of Parkinson's disease, with particular emphasis on bradykinesia and rigidity, masked facies, and resting tremor, irrespective of concurrent Fragile X‐associated syndromic or non‐syndromic neurological manifestations. In the Australian cohort, diagnostic confirmation required Unified Parkinson's Disease Rating Scale (UPDRS) scores exceeding one standard deviation above normative values [21, 22]. The Mini‐Mental State Examination (MMSE) [23] was utilized to screen for cognitive impairment. At the same time, psychiatric symptomatology was quantified using anxiety and depression subscale scores derived from the Symptom Checklist‐90‐Revised (SCL‐90‐R) instrument [24]. The choice of individual features for analysis of this combined sample was restricted to those assessed by the same tests at both Australian and American sites (with the exception of the UPDRS used as an additional diagnostic criterion in the Australian sample).

### 2.4 CGG Repeat Size and FMR1 mRNA Expression Levels

CGG repeat allele sizing for premutation status determination and *quantification of FMR1 mRNA expression* were performed for both study cohorts at the Laboratory of Dr. Tassone, MIND Institute, University of California Davis Medical Center, Sacramento, CA, USA. Genomic DNA was extracted from peripheral blood lymphocytes using standardized protocols with a Puregene Kit (Gentra Inc., Minneapolis, MN, USA). Methylation status and CGG allele sizing and Activation ratio in females were determined through combined Southern blotting and polymerase chain reaction (PCR) analyses following previously established methodologies [25].

Total RNA isolation was performed from 3 mL of whole blood collected in PAX gene collection tubes (Qiagen, Valencia, CA, USA). *FMR1* mRNA expression levels were measured using qRT‐PCR using Assays‐On‐Demand (Applied Biosystems, Foster City, CA, USA) and custom TaqMan primers and probe assays according to previously validated protocols [26].

### 2.5 Statistical Analysis

All statistical analyses were conducted using Stata Statistical Software version 19 (StataCorp LLC, College Station, TX, USA). The primary independent variables were the CGG repeat length, age, and *FMR1* mRNA expression levels. Univariate associations between each independent molecular variable and clinical outcome were assessed using independent sample *t*‐tests. Multivariate relationships were evaluated using *F*‐tests within an appropriate regression framework.

Logistic regression models were employed to examine the predictive value of the independent variables for dichotomous outcomes, including FXTAS diagnosis, parkinsonism, anxiety, and depression. Multinomial logistic regression was used to assess the associations with clinical phenotype classifications (subclinical, monosymptomatic, and syndromic). Linear regression models were applied to evaluate predictors of continuous cognitive performance, as indexed by the MMSE score. All analyses conducted in the combined sample were stratified by sex.

## 3 Results

### 3.1 Sample Characteristics

Of the initial cohort of 241 participants, only 176 cases with all the essential data points available were included in the analysis. These individuals were classified into three clinical phenotypes as specified in the Methods section: asymptomatic, non‐syndromic, and FXTAS, which occurred in 23.9%, 33.0%, and 43.2%, respectively, in the total sample. Baseline demographic, clinical, and molecular characteristics, listed in Table 1 for total, female, and male samples, significantly differed between these clinical categories, except for the Depression and Anxiety scores. It is interesting to note that the age for the non‐syndromic category was not lower than for the FXTAS group in both total and female samples (and was in the same age bracket for the male sample), which supports evidence that this category does not necessarily, or often, evolve into FXTAS with progressing age.

**TABLE 1:** Demographic, clinical, and molecular characterization of the combined cohort of 176 male and female premutation carriers.

Clinical categories	Asymptomatic	Non‐syndromic	FXTAS	Total
**Total sample (*N* = 176)**	42 (23.9%)	58 (33.0%)	76 (43.2%)	176 (100%)
Age	54.62 (9.03)	67.71 (7.60)	66.80 (6.81)	64.19 (9.32)
mRNA	2.15 (0.59)	2.33 (0.69)	2.68 (0.63)	2.43 (0.67)
CGG	83.00 (16.03)	84.63 (16.46)	96.72 (11.91)	89.49 (15.79)
Parkinsonism	0.032 (0.180)	0.20 (0.40)	0.28 (0.45)	0.20 (0.40)
MMSE	29.19 (1.56)	28.26 (2.34)	27.72 (2.80)	28.23 (2.47)
Depression	57.39 (7.82)	57.63 (8.67)	59.89 (10.47)	58.50 (9.26)
Anxiety	53.39 (9.24)	55.47 (10.42)	56.68 (8.94)	55.43 (9.55)
**Males (*N* = 111)**	16 (38.1%)	27 (46.6%)	68 (89.5%)	111 (100%)
Age	56.19 (11.02)	66.63 (7.53)	67.37 (5.89)	65.58 (8.13)
mRNA	2.10 (0.81)	2.46 (0.85)	2.70 (0.64)	2.55 (0.75)
CGG	81.07 (20.83)	84.58 (19.27)	96.67 (12.36)	91.59 (16.77)
Parkinsonism	0.00 (0.00)	0.31 (0.47)	0.29 (0.46)	0.26 (0.44)
MMSE	28.91 (2.30)	28.01 (2.83)	27.60 (2.92)	27.88 (2.83)
Depression	55.50 (8.98)	56.22 (10.76)	59.78 (10.67)	58.34 (10.50)
Anxiety	53.25 (7.78)	52.28 (9.84)	56.08 (8.75)	54.80 (8.92)
**Females (*N* = 65)**	26 (40.0%)	31 (47.7%)	8 (12.3%)	65 (100.0%)
Age	53.65 (7.63)	68.65 (7.66)	62.00 (11.64)	61.83 (10.71)
mRNA	2.18 (0.42)	2.19 (0.45)	2.51 (0.47)	2.22 (0.44)
CGG	84.12 (12.83)	84.68 (14.01)	97.13 (7.66)	85.99 (13.43)
Parkinsonism	0.05 (0.23)	0.10 (0.31)	0.17 (0.41)	0.09 (0.29)
MMSE	29.33 (1.11)	28.41 (1.88)	28.63 (1.60)	28.78 (1.63)
Depression	58.48 (7.08)	58.64 (6.84)	61.67 (7.51)	58.76 (6.87)
Anxiety	53.48 (10.16)	57.76 (10.41)	66.67 (6.66)	56.47 (10.50)

Predictably, the proportion of females was significantly higher in the unaffected category and lower in the FXTAS category compared to males. But our novel and somewhat unexpected finding was that approximately one‐third of both male and female carriers belonged to the non‐syndromic category. The frequency of Parkinsonism increased more rapidly with the severity of clinical involvement, but cognitive performance, as indexed by the Mini‐Mental Score (MMSE), showed only minimal decline in the male sample. A parallel trend was observed between the CGG repeat size and *FMR1* mRNA expression levels, but the rapid increase in the mean CGG repeat length in the FXTAS group is noteworthy.

### 3.2 Genotype–Phenotype Relationships

**FIGURE 1:**
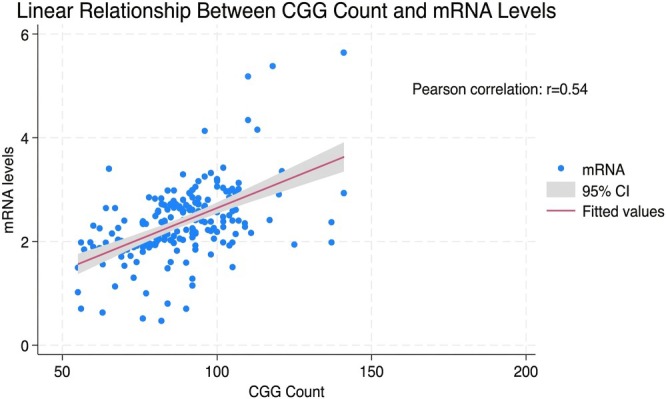
Scatter graph showing messenger RNA expression levels on different CCG counts.

As expected, the two crucial *FMR1* genomic markers were highly intercorrelated (Figure 1), with the CGG repeat allele size being positively associated with *FMR1* mRNA expression levels (β = 0.02, *p* < 0.001). The regression lines representing the model‐predicted values were consistent across estimations. Although the association was generally linear, the variance in mRNA expression levels and overall variability dramatically increased at higher CGG repeat sizes, with some individuals showing exceptionally high expression levels (> 4.5) for CGG repeat > 100. Overall, considering the value of Pearson correlation between these two biomarkers of 0.54, the contribution to their total variance does not exceed a modest 0.29.

**FIGURE 2:**
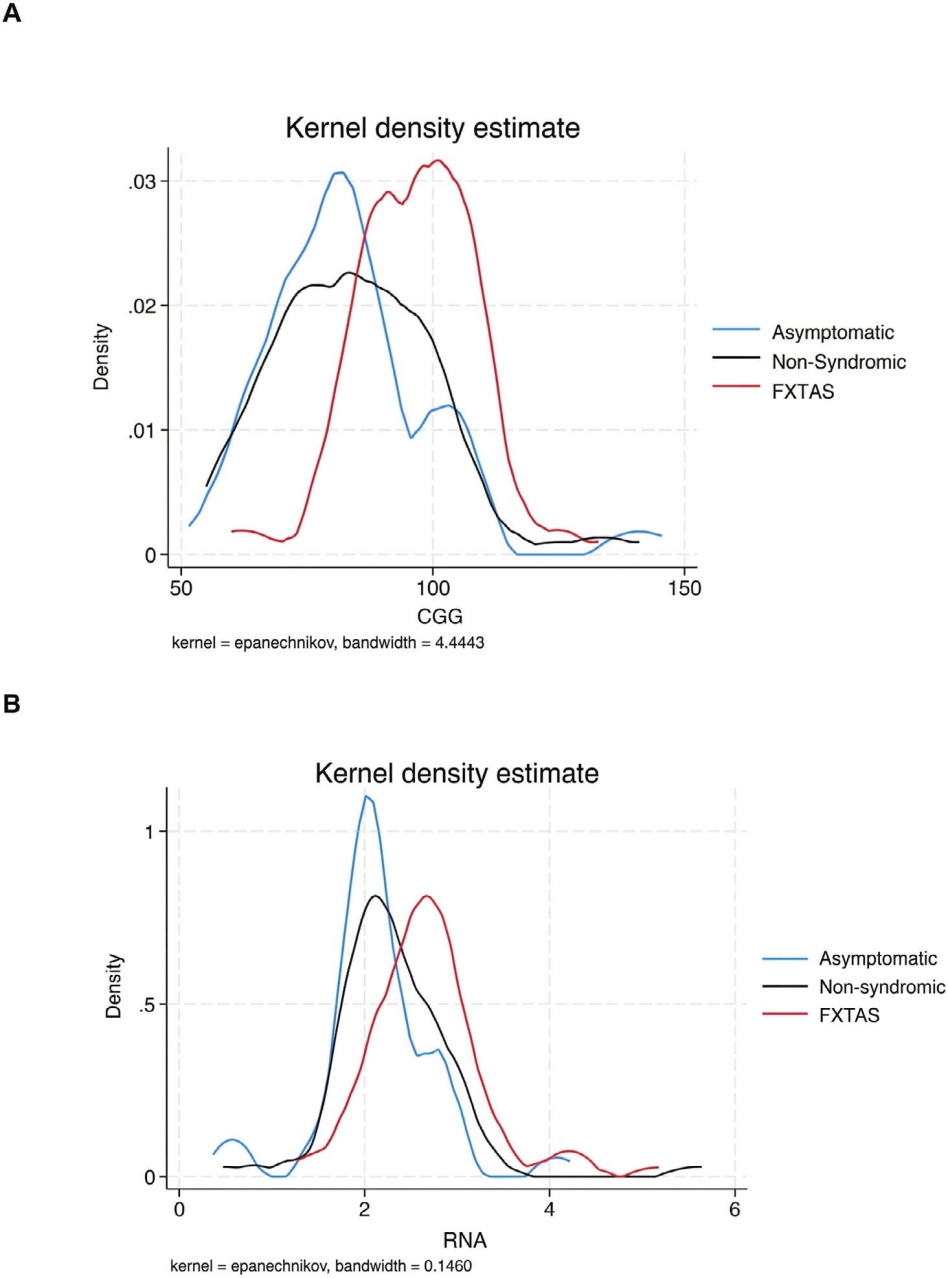
Kernel density estimate of CGG repeat number (A) and mRNA expression levels (B) in each of the three clinical categories.

Kernel density estimates for CGG repeat expansion size corresponding to the clinical severity categories are shown in Figure 2A. The asymptomatic distribution is left‐shifted, peaking near 75 repeats, whereas FXTAS cases show a broader, right‐shifted distribution peaking around 100 repeats. Notably, the distribution for the non‐syndromic group is also distinctive, but it shows substantial overlap with the distributions of the two extreme groups. This trend is more evident for the mRNA distribution shown in Figure 2B. These findings support a dimensional interpretation of FXTAS symptom severity rather than a dichotomous classification.

These distributions are largely similar between the two sexes, apart from one notable difference illustrated in the Supplement Figures, which show Kernel density estimates for both CGG repeat size and mRNA levels against three clinical categories separately for males (Figure S1) and females (Figure S2). A shift of the curve for the non‐syndromic category towards the higher values of both CGG repeats and mRNA is more pronounced in the female sample, which might be accounted for by the sex difference in penetrance linked to the effect of the second (normal) X chromosome and possibly some other preventative factors resulting in a much lower rate of transition of non‐syndromic to FXTAS forms in females compared with male carriers.

Table 2 summarizes the results of the regression analyses examining CGG repeat size, age, and mRNA expression levels as predictors across several clinical outcomes stratified by sex (M = male, F = female, and C = combined). When FXTAS status was treated as a binary variable, age was a consistent predictor across strata, with CGG repeat size and mRNA expression levels reaching significance in males and the combined group. However, when the clinical categories (asymptomatic, non‐syndromic, and FXTAS) were modeled using multinomial regression, all three predictors were statistically significant across all strata. The occurrence of Parkinsonism was predicted by CGG repeat size and age. MMSE scores were significantly and negatively correlated with age in males, females, and the combined sample, as well as with CGG repeat size in males only. Depression scores were associated with age in females and in the combined group, while anxiety scores were negatively correlated with age in the combined group.

**TABLE 2:** Summary of results from appropriate regression models for all the outcome (clinical) variables against genetic predictors: CGG expansion size and mRNA expression levels, including age as a modifier.

Outcome	CGG	Age	mRNA	Combined model (CGG plus mRNA plus age)
FXTAS vs. Non‐FXTAS	+ (M,C)	+ (M, F, C)	+ (M, C)	+ (M,C)
Clinical Categories Ranked	+ (M, F, C)	+ (M, F, C)	+ (M, F, C)	+ (M, F, C)
Parkinsonism	+ (C)	+ (M, F, C)	NS	+(M, C)
Mini‐Mental Score	NS	− (M, F, C)	NS	− (M, C)
Depression Score	NS	+ (F, C)	NS	+ (C)
Anxiety Score	NS	+ (C)	NS	NS

Abbreviations: −, negative relationship; +, positive relationship; C, sexes combined; F, Females; M, males; NS, not significant.

The original results from univariate and multivariate (with age and sex included as modifiers) regressions for two binomial outcomes: FXTAS and Parkinsonism versus both, CGG expansion size and mRNA genetic predictors, are shown in Table 3. Apart from the overwhelming effects of sex and age on both these outcomes, the less predictable relationships are between Parkinsonism and CGG expansion size, which still borderlines significance after age and sex adjustment in multivariate analysis (see confidence intervals).

**TABLE 3:** Results of univariate and multivariate regression, including FXTAS and Parkinsonism binomial outcome variables versus age, sex, and both genetic predictors based on the combined cohort of *FMR1* premutation carriers.

	UNIVARIATE OR	*p*	95% CI	MULTIVARIATE aOR	*p*	95% CI
**FXTAS** ^**a**^						
age	1.16	< 0.001	1.11, 1.21	1.14	0.000	1.09, 1.20
sex	2.37	0.004	1.32, 4.28	1.66	0.238	0.71, 3.88
mRNA	2.27	0.003	1.33, 3.88	2.22	0.04	1.04, 4.76
cgg	1.03	< 0.001	1.01, 1.05	1.02	0.168	0.99, 1.05
**PARKINSONISM** ^**a**^						
age	1.05	0.006	1.02, 1.10	1.06	0.02	1.01, 1.11
sex	2.56	0.02	1.15, 5.68	3.18	0.02	1.18, 8.60
mRNA	1.24	0.36	0.78, 1.94	0.82	0.524	0.45, 1.50
cgg	1.02	0.03	1.00, 1.04	1.03	0.079	1.00, 1.05

^a^ Denotes Odds Ratio calculated after logistic regression.

The results of robust regression, exemplifying a model with diagnosable FXTAS considered as part of the spectrum encompassing lesser neurological involvement categories versus CGG expansion size and RNA, are shown in Table 4. If both genetic predictors are included, the effect of mRNA across the spectrum is evident.

**TABLE 4:** Robust regression considering neurological involvement ranked from asymptomatic to syndromic FXTAS as outcome variables versus age and both genetic predictors based on the combined cohort of *FMR1* premutation carriers.

Clinical category	Coefficient	Robust SE	`*z*‐score	*p* > |*z*|	95% CI
Asymptomatic	(base outcome)				
Non syndromic					
Age	0.201	0.037	5.47	0.000	0.128, 0.272
CGG	−0.015	0.024	−0.61	0.544	−0.062, 0.033
mRNA	1.417	0.664	2.13	0.033	0.116, 2.717
_cons	−13.940	2.944	−4.73	0.00	−19.711, −8.169
FXTAS					
Age	0.216	0.039	5.53	0.000	0.140, 0.293
CGG	0.049	0.030	1.65	0.099	−0.009, 0.107
mRNA	1.587	0.607	2.61	0.009	0.398, 2.777
_cons	−20.788	3.586	−5.80	0.00	−27.817, −13.759

## 4 Discussion

In this study, we explored the relationships between neurological, cognitive, and psychiatric changes attributed to FXTAS and the underlying *FMR1* gene's markers: CCG repeat number and mRNA expression levels in a sample of 176 male and female carriers of premutation alleles. We considered FXTAS diagnosed using all the established criteria [9] as a discrete category; alternatively, as a distinctive syndromic form within the broader spectrum of non‐syndromic neural involvements [19]. The major finding from regression analysis showed that the most consistent relationship was between a clear continuum of severity of clinical manifestations with FXTAS representing the most severe form of involvement, and both genetic markers, while correlations between the syndromic form considered individually were not significant for either CGG or mRNA in the female sample. These results suggest the common link, through genetic underpinning, between (potentially evolving) non‐syndromic and syndromic forms of manifestation, with the final age‐related outcome determined by the number of other factors still to be discovered. This concept is illustrated in Figure 2, where the distributions of CGG repeat/mRNA expression levels in carriers are distinctive but also overlap with those of both FXTAS and asymptomatic individuals, respectively. This is consistent with clinical data showing that some proportion of these carriers does not evolve beyond a non‐syndromic form.

Although the concept of a continuum of neural involvement rather than discrete FXTAS/non‐FXTAS categories is still not fully recognized at the clinical level, the most recent longitudinal study of neuroradiological features revealed reduced brain volumes in both the non‐FXTAS and FXTAS groups, with the condition progressing over time [27].

Notably, in another study, nearly half of premutation females were not aware of having tremor (as shown by CATSYS results [28]) or postural instability [29]. A significance and potential predictive role of these isolated prodromal features has been emphasized by revealing close correlations between isolated (kinetic) tremor and mild executive function impairment in another sample of the non‐FXTAS female carriers [12].

Supportive of clinical data, the existence of neuropathological or metabolic changes in both male and female carriers has already been established [6, 30, 31, 32, 33, 34, 35, 36, 37, 38]. A more recent study, showing a decline in mitochondrial activity in ‘pre‐symptomatic’ female premutation carriers, led to the conclusion that the neural involvement with progressing age in these carriers could be linked to a lifetime accumulation of cellular damage [38].

The above data clearly suggest the need to identify clinical or other markers that are most predictive of clinical outcome with advancing age and could thus help in assessing the risk of progression towards syndromic FXTAS. Long‐term follow‐up studies would also allow the determination of the true proportion of these carriers in whom the subclinical manifestations become symptomatic or prodromal of syndromic FXTAS. The notion of a broad spectrum of neural involvement encompassing manifestations insufficient to formally diagnose FXTAS at the time of examination has been the first step in this direction [1, 11, 13, 14]. The present results have provided novel, compelling evidence from the genotype–phenotype relationship patterns.

Important data towards a broader understanding of the concept and the dynamics of non‐syndromic cases have been provided by our earlier longitudinal study of 57 apparently asymptomatic female premutation carriers who underwent neurological examinations over a 10‐year period. It is remarkable that 19 of those females had a significant elevation in at least one motor score at the initial examination, but only six of them showed further marked elevation of these scores over time, with half evolving into a diagnosable FXTAS after nine to ten years ([11] and unpublished data).

From a clinical perspective, an accurate assessment of the risk of FXTAS based on the relevant markers is of high importance to many fragile X families, especially those female carriers who care for their affected fathers and are aware of the severity of this condition that progresses to death. Consequently, for such individuals who may be asymptomatic or who have an isolated tremor, the diagnosis of FXTAS would not be either correct or appropriate. Therefore, to appreciate the continuum, but not overwhelm the patient, the term ‘preFXTAS’ has been proposed for individuals presenting with FXTAS‐like features but not meeting full FXTAS diagnostic criteria [13]. This term, however, is still contentious considering the existing evidence that only a small proportion of such low‐symptomatic female carriers evolve into a diagnosable FXTAS.

Apart from the core clinical manifestations of FXTAS, the present study explored the correlation of *FMR1* genotype with several minor clinical features of this syndrome, including Parkinsonism, general cognitive impairment and, still controversial, mood disorders: depression and anxiety. Notably, in our study, a dichotomous score for parkinsonism showed significant associations with CGG repeat, as well as with age, in both combined and male samples. However, the presence of clinical overlap between premutation‐related neurological changes, especially in severe form of FXTAS, and Parkinsonism such as tandem walking and some other measures of cerebellar ataxia, or in certain types of tremor, makes it difficult to interpret this finding, and may account for a wide discrepancy in the reported prevalence of parkinsonism in premutation carriers with FXTAS varying from 29% to 60% [39].

Although the reason for this comorbidity is not yet fully understood, the results of neuropathological examination in deceased patients diagnosed with both FXTAS and PD revealed more than one underlying process, leading the authors to the conclusion that ‘*FMR1* should be recognized as one of the exceptional genetic causes of Parkinsonism with typical presynaptic dopaminergic loss and LBs’ [39]. Notably, in other examples of concomitant diagnosis [40], CGG expansion was at the lowest end of the distribution, that is, in a ‘gray zone’ category, which had already been shown as one of the PD genetic risk factors [41].

There may be other mechanisms leading to the occurrence of Parkinsonian features as part of a diagnosable FXTAS. A genetic link between premutation‐related neurological changes and parkinsonism was suggested in our earlier reports [19, 42]. Niu et al. [43] found close correlations between bradykinesia (scored as part of the UPDRS scale) and both CGG expansion size and *FMR1 mRNA* expression levels, which implicated pathophysiological mechanisms linking the mRNA toxicity, dopamine pathway, and parkinsonism in FXTAS. Mitochondrial dysfunction, increasing vulnerability to diverse neural involvement, may represent another shared feature of PD and FXTAS [44, 45, 46, 47, 48].

Cognitive impairment is another minor diagnostic trait in FXTAS, with more prevalent executive dysfunction extending beyond a syndromic form [18, 49, 50]. Despite using MMSE (which was the only cognitive test available to us in both cohorts), this score did show a significant age‐independent association with the CGG expansion size in the combined sample. In contrast, depression and anxiety scores did not show any relationships with the *FMR1* genotype, suggesting that these traits may be influenced by other factors more effective than the premutation genotype and thus require individualized treatment and prevention.

The combined model applied in the present study incorporated *FMR1* mRNA expression levels, as well as the CGG expansion size and age as predictors. These three factors showed significant associations across multiple clinical outcomes, but most consistently in all samples and for both genetic predictors, in the hierarchical ranking of the clinical severity model. While comparing the FXTAS model to the clinical categories model in regression, the association with mRNA was not significant in the female sample in the former. Overall, individuals in the asymptomatic group had the lowest mRNA levels (2.15 ± 0.59), followed by non‐syndromic (2.33 ± 0.69) and syndromic patients (2.68 ± 0.63) (*p* < 0.001). This parallel is not unexpected considering that elevation of ‘toxic’ mRNA, which causes the sequestration and functional perturbation of CGG binding proteins, leads to cumulative oxidative stress and progressive neuronal damage [2]. On the other hand, we have also noted some discrepancy between the effect of CGG repeat size and mRNA expression level on the phenotype, with RNA appearing to be a bigger driver of the effect than CGG expansions. This is not unexpected considering the results of some studies, including our own observations that the RNA expression level and therefore toxicity can be different even for alleles of the same size, with some still undisclosed factors possibly affecting the expression. CGG repeat instability that we have earlier reported in premutation carriers [51] may be one of such factors accounting for this discrepancy which, in our data, is most noticeable in the upper and also the lower end of the CGG distribution (as in Figure 1).

This study has several limitations. Our convenience sampling approach may have introduced a selection bias, as we likely oversampled individuals with a more severe form of the disease and those with a family history of fragile X‐associated conditions. Second, because our analysis combined samples from two different investigative sites, variability in the classification of neurological involvement across cohorts may have affected the accuracy of clinical rankings or diagnostic categories. However, the relatively large sample size provided sufficient power to detect a significant trend in the data. Although the unequal sex distribution reduced the statistical power of the sex‐specific analyses, this was not within the scope of this study.

Instead, the present findings have supported a paradigm shift towards recognizing a continuum of involvement, with molecular genetic changes and potential neurotoxic consequences of CGG repeat expansion and mRNA elevation concerning the carriers who do not necessarily meet strict FXTAS criteria. This clearly implies that binary classification may overlook non‐syndromic (especially subclinical) manifestations, which should be recognized in risk estimates and in individualized therapeutic strategies, in contrast to rigid categorical treatment models that are likely to overlook clinically meaningful subthreshold changes.

## Author Contributions

This study was conceptualized by D.Z.L., who also collected clinical neurological data jointly with E.St. in the Australian sample. Psychological/psychiatric data were collected by A.A. under the supervision of E.St. R.J.H. collected neurological data and coordinated/supervised E.Sa. in clinical and neuropsychiatric data collection in the US sample. F.T. performed laboratory analysis on both samples. F.C. performed statistical analysis under the supervision of M.E.R. and N.G.M. and contributed to statistical/results sections of the paper written by D.Z.L. and F.T., with editorial contributions from R.J.H., M.E.R., and N.G.M.

## Funding

Data collection for this study was supported by the National Institutes of Child Health and Human Development Grant, US, No HD 036071, to Dr. DZ Loesch, Dr. F. Tassone, and Prof. RJ Hagerman; an NICHD grant for the MIND Institute IDDRC P50 HD103526; Rebecca L. Cooper Medical Research Foundation through an Al & Val Rosenstrauss Fellowship (F20231230) to Dr. M. Renteria; National Health and Medical Research Council Australia project grant No CF06/0269 to Prof. E. Storey, Dr. D.Z. Loesch, and Dr. F. Tassone; and an NHMRC Investigator Grant (APP1172990) to Prof. N.G. Martin. Additionally, another National Health and Medical Research Council Australia project grant, No CF06/0269, was awarded to Prof. E. Storey, Dr. D. Z. Loesch, and Dr. F. Tassone.

## Conflicts of Interest

The authors declare no conflicts of interest.

## Supporting information


**Data S1:** Kernel density estimate of CGG repeat number (a) and mRNA expression levels (b) in each of the three clinical categories for the male sample.

**Data S2:** Kernel density estimate of mRNA expression levels (a) and CGG repeat number (b) in each of the three clinical categories for the female sample.

## Data Availability

Data supporting the findings of this study are available from the authors upon reasonable request.

## References

[1] D. Loesch and R. Hagerman , “Unstable Mutations in the FMR1 Gene and the Phenotypes,” Advances in Experimental Medicine and Biology 769 (2012): 78–114. 23560306 10.1007/978-1-4614-5434-2_6 PMC4124039

[2] F. Tassone , D. Protic , E. G. Allen , et al., “Insight and Recommendations for Fragile X‐Premutation‐Associated Conditions From the Fifth International Conference on FMR1 Premutation,” Cells 12, no. 18 (2023): 2330. 37759552 10.3390/cells12182330 PMC10529056

[3] R. J. Hagerman , M. Leehey , W. Heinrichs , et al., “Intention Tremor, Parkinsonism, and Generalized Brain Atrophy in Male Carriers of Fragile X,” Neurology 57, no. 1 (2001): 127–130. 11445641 10.1212/wnl.57.1.127

[4] S. Jacquemont , R. J. Hagerman , M. A. Leehey , et al., “Penetrance of the Fragile X–Associated Tremor/Ataxia Syndrome in a Premutation Carrier Population,” Journal of the American Medical Association 291, no. 4 (2004): 460–469. 14747503 10.1001/jama.291.4.460

[5] P. J. Hagerman and R. J. Hagerman , “Fragile X‐Associated Tremor/Ataxia Syndrome,” Annals of the New York Academy of Sciences 1338, no. 1 (2015): 58–70. 25622649 10.1111/nyas.12693 PMC4363162

[6] F. Tassone , C. M. Greco , M. R. Hunsaker , et al., “Neuropathological, Clinical and Molecular Pathology in Female Fragile X Premutation Carriers With and Without FXTAS,” Genes, Brain, and Behavior 11, no. 5 (2012): 577–585. 22463693 10.1111/j.1601-183X.2012.00779.x PMC3965773

[7] F. Tassone , I. Christine , and P. J. Hagerman , “FMR1 RNA Within the Intranuclear Inclusions of Fragile X‐Associated Tremor/Ataxia Syndrome (FXTAS),” RNA Biology 1, no. 2 (2004): 103–105. 17179750 10.4161/rna.1.2.1035

[8] A. M. Cabal‐Herrera , N. Tassanakijpanich , M. J. Salcedo‐Arellano , and R. J. Hagerman , “Fragile X‐Associated Tremor/Ataxia Syndrome (FXTAS): Pathophysiology and Clinical Implications,” International Journal of Molecular Sciences 21, no. 12 (2020): 4391. 32575683 10.3390/ijms21124391 PMC7352421

[9] D. A. Hall , R. C. Birch , M. Anheim , et al., “Emerging Topics in FXTAS,” Journal of Neurodevelopmental Disorders 6, no. 1 (2014): 31. 25642984 10.1186/1866-1955-6-31 PMC4141265

[10] D. Loesch , A. Churchyard , P. Brotchie , M. Marot , and F. Tassone , “Evidence for, and a Spectrum of, Neurological Involvement in Carriers of the Fragile X Pre‐Mutation: FXTAS and Beyond,” Clinical Genetics 67, no. 5 (2005): 412–417. 15811008 10.1111/j.1399-0004.2005.00425.x

[11] D. Z. Loesch , F. Tassone , A. Atkinson , et al., “Differential Progression of Motor Dysfunction Between Male and Female Fragile X Premutation Carriers Reveals Novel Aspects of Sex‐Specific Neural Involvement,” Frontiers in Molecular Biosciences 7 (2021): 577246. 33511153 10.3389/fmolb.2020.577246 PMC7835843

[12] D. Z. Loesch , A. Atkinson , D. A. Hall , F. Tassone , P. Stimpson , and E. Storey , “Cognitive Status Correlates of Subclinical Action Tremor in Female Carriers of FMR1 Premutation,” Frontiers in Neurology 15 (2024): 1401286. 38903175 10.3389/fneur.2024.1401286 PMC11188871

[13] V. Liani , C. Torrents , E. Rolleri , et al., “Premutation Females With preFXTAS,” International Journal of Molecular Sciences 26, no. 6 (2025): 2825. 40141467 10.3390/ijms26062825 PMC11942631

[14] E. G. Allen , M. A. Leehey , F. Tassone , and S. Sherman , “Genotype/Phenotype Relationships in FXTAS,” in FXTAS, FXPOI, and Other Premutation Disorders, 2nd ed., ed. F. Tassone and D. A. Hall (Springer International Publishing, 2016), 129–160.

[15] F. Tassone , J. Adams , E. M. Berry‐Kravis , et al., “CGG Repeat Length Correlates With Age of Onset of Motor Signs of the Fragile X‐Associated Tremor/Ataxia Syndrome (FXTAS),” American Journal of Medical Genetics Part B: Neuropsychiatric Genetics 144B, no. 4 (2007): 566–569. 10.1002/ajmg.b.30482 17427188

[16] M. A. Leehey , E. Berry‐Kravis , C. G. Goetz , et al., “FMR1 CGG Repeat Length Predicts Motor Dysfunction in Premutation Carriers,” Neurology 70, no. 16 Pt 2 (2008): 1397–1402. 18057320 10.1212/01.wnl.0000281692.98200.f5 PMC2685188

[17] D. Hessl , F. Tassone , D. Z. Loesch , et al., “Abnormal Elevation of FMR1 mRNA Is Associated With Psychological Symptoms in Individuals With the Fragile X Premutation,” American Journal of Medical Genetics Part B: Neuropsychiatric Genetics 139B, no. 1 (2005): 115–121. 10.1002/ajmg.b.30241 16184602

[18] J. Grigsby , A. G. Brega , S. Jacquemont , et al., “Impairment in the Cognitive Functioning of Men With Fragile X‐Associated Tremor/Ataxia Syndrome (FXTAS),” Journal of the Neurological Sciences 248, no. 1 (2006): 227–233. 16780889 10.1016/j.jns.2006.05.016

[19] D. Z. Loesch , F. Chafota , M. Q. Bui , et al., “Parkinson's Disease Polygenic Risk Score and Neurological Involvement in Carriers of the FMR1 Premutation Allele: A Case for Genetic Modifier,” Molecular Genetics & Genomic Medicine 12, no. 11 (2024): e70043. 39588919 10.1002/mgg3.70043 PMC11590032

[20] P. J. Hagerman and R. J. Hagerman , “The Fragile‐X Premutation: A Maturing Perspective,” American Journal of Human Genetics 74, no. 5 (2004): 805–816. 15052536 10.1086/386296 PMC1181976

[21] S. Fahn , R. Elton , and UPDRS Development Committee , “The Unified Parkinson's Disease Rating Scale,” in Recent Developments in Parkinson's Disease, 2nd ed., ed. S. Fahn , C. D. Marsden , D. B. Calne , and M. Goldstein (Macmillan Healthcare Information, 1987), 153–163, 293‐304.

[22] Movement Disorder Society Task Force on Rating Scales for Parkinson's Disease , “The Unified Parkinson's Disease Rating Scale (UPDRS): Status and Recommendations,” Movement Disorders 18, no. 7 (2003): 738–750. 12815652 10.1002/mds.10473

[23] M. F. Folstein , S. E. Folstein , and P. R. McHugh , “‘Mini‐Mental State’: A Practical Method for Grading the Cognitive State of Patients for the Clinician,” Journal of Psychiatric Research 12, no. 3 (1975): 189–198. 1202204 10.1016/0022-3956(75)90026-6

[24] L. Derogatis , SCL‐90‐R: Symptom Checklist‐90‐R. Administration, Scoring and Procedures Manual, 3rd ed. (NCS Pearson, 1994).

[25] S. Filipovic‐Sadic , S. Sah , L. Chen , et al., “A Novel FMR1 PCR Method for the Routine Detection of Low Abundance Expanded Alleles and Full Mutations in Fragile X Syndrome,” Clinical Chemistry 56, no. 3 (2010): 399–408. 20056738 10.1373/clinchem.2009.136101 PMC4031651

[26] F. Tassone , R. J. Hagerman , D. Garcia‐Arocena , E. Khandjian , C. Greco , and P. J. Hagerman , “Intranuclear Inclusions in Neural Cells With Premutation Alleles in Fragile X Associated Tremor/Ataxia Syndrome,” Journal of Medical Genetics 41, no. 4 (2004): e43. 15060119 10.1136/jmg.2003.012518 PMC1735735

[27] D. Hessl , J. Y. Wang , G. Espinal , et al., “Longitudinal Analysis of Neuroradiological Biomarkers for Fragile X‐Associated Tremor/Ataxia Syndrome and Implications for Clinical Trials,” Annals of Neurology 98 (2025): 471–481. 40459253 10.1002/ana.27267 PMC12392051

[28] J. L. Juncos , J. T. Lazarus , E. Graves‐Allen , et al., “New Clinical Findings in the Fragile X‐Associated Tremor Ataxia Syndrome (FXTAS),” Neurogenetics 12, no. 2 (2011): 123–135. 21279400 10.1007/s10048-010-0270-5 PMC3766636

[29] C. O'Keeffe , L. P. Taboada , N. Feerick , L. Gallagher , T. Lynch , and R. B. Reilly , “Complexity Based Measures of Postural Stability Provide Novel Evidence of Functional Decline in Fragile X Premutation Carriers,” Journal of Neuroengineering and Rehabilitation 16, no. 1 (2019): 87. 31299981 10.1186/s12984-019-0560-6 PMC6624948

[30] J. Y. Wang , D. Hessl , R. J. Hagerman , F. Tassone , and S. M. Rivera , “Age‐Dependent Structural Connectivity Effects in Fragile × Premutation,” Archives of Neurology 69, no. 4 (2012): 482–489. 22491193 10.1001/archneurol.2011.2023 PMC3979438

[31] J. Y. Wang , D. Hessl , A. Schneider , F. Tassone , R. J. Hagerman , and S. M. Rivera , “Fragile X–Associated Tremor/Ataxia Syndrome: Influence of the FMR1 Gene on Motor Fiber Tracts in Males With Normal and Premutation AllelesFragile X–Associated Tremor/Ataxia Syndrome,” JAMA Neurology 70, no. 8 (2013): 1022–1029. 23753897 10.1001/jamaneurol.2013.2934 PMC4028037

[32] G. Battistella , J. Niederhauser , E. Fornari , et al., “Brain Structure in Asymptomatic FMR1 Premutation Carriers at Risk for Fragile X‐Associated Tremor/Ataxia Syndrome,” Neurobiology of Aging 34, no. 6 (2013): 1700–1707. 23298734 10.1016/j.neurobiolaging.2012.12.001

[33] A. L. Shelton , K. M. Cornish , C. M. Kraan , R. Lozano , M. Bui , and J. Fielding , “Executive Dysfunction in Female FMR1 Premutation Carriers,” Cerebellum 15, no. 5 (2016): 565–569. 27126308 10.1007/s12311-016-0782-0

[34] D. Z. Loesch , S. J. Annesley , N. Trost , et al., “Novel Blood Biomarkers Are Associated With White Matter Lesions in Fragile X‐ Associated Tremor/Ataxia Syndrome,” Neurodegenerative Diseases 17, no. 1 (2017): 22–30. 27602566 10.1159/000446803 PMC10964908

[35] D. Z. Loesch , N. Trost , M. Q. Bui , et al., “The Spectrum of Neurological and White Matter Changes and Premutation Status Categories of Older Male Carriers of the FMR1 Alleles Are Linked to Genetic (CGG and FMR1 mRNA) and Cellular Stress (AMPK) Markers,” Frontiers in Genetics 9 (2018): 531. 30483310 10.3389/fgene.2018.00531 PMC6241173

[36] J. Y. Wang , D. Hessl , R. J. Hagerman , et al., “Abnormal Trajectories in Cerebellum and Brainstem Volumes in Carriers of the Fragile X Premutation,” Neurobiology of Aging 55 (2017): 11–19. 28391068 10.1016/j.neurobiolaging.2017.03.018 PMC5498112

[37] D. R. Hocking , D. Z. Loesch , N. Trost , et al., “Total and Regional White Matter Lesions Are Correlated With Motor and Cognitive Impairments in Carriers of the FMR1 Premutation,” Frontiers in Neurology 10, no. 832 (2019): 832. 31456732 10.3389/fneur.2019.00832 PMC6700239

[38] E. Napoli , Y. A. McLennan , A. Schneider , F. Tassone , R. J. Hagerman , and C. Giulivi , “Characterization of the Metabolic, Clinical and Neuropsychological Phenotype of Female Carriers of the Premutation in the X‐Linked FMR1 Gene,” Frontiers in Molecular Biosciences 7 (2020): 578640. 33195422 10.3389/fmolb.2020.578640 PMC7642626

[39] M. J. Salcedo‐Arellano , M. W. Wolf‐Ochoa , T. Hong , et al., “Parkinsonism Versus Concomitant Parkinson's Disease in Fragile X–Associated Tremor/Ataxia Syndrome,” Movement Disorders Clinical Practice 7, no. 4 (2020): 413–418. 32373658 10.1002/mdc3.12942 PMC7197312

[40] E. De Pablo‐Fernandez , K. M. Doherty , J. L. Holton , et al., “Concomitant Fragile X‐Associated Tremor Ataxia Syndrome and Parkinson's Disease: A Clinicopathological Report of Two Cases,” Journal of Neurology, Neurosurgery & Psychiatry 86, no. 8 (2015): 934. 25476004 10.1136/jnnp-2014-309460 PMC4516009

[41] D. Loesch , F. Tassone , J. Lo , et al., “New Evidence for, and Challenges in, Linking Small CGG Repeat Expansion FMR1 Alleles With Parkinson's Disease,” Clinical Genetics 84, no. 4 (2013): 382–385. 23198693 10.1111/cge.12070

[42] D. Z. Loesch , D. L. Duffy , N. G. Martin , F. Tassone , A. Atkinson , and E. Storey , “'Essential Tremor' Phenotype in FMR1 Premutation/Gray Zone Sibling Series: Exploring Possible Genetic Modifiers,” Twin Research and Human Genetics 24, no. 2 (2021): 95–102. 33757613 10.1017/thg.2021.10

[43] Y.‐Q. Niu , J.‐C. Yang , D. A. Hall , et al., “Parkinsonism in Fragile X‐Associated Tremor/Ataxia Syndrome (FXTAS): Revisited,” Parkinsonism & Related Disorders 20, no. 4 (2014): 456–459. 24491663 10.1016/j.parkreldis.2014.01.006 PMC4019503

[44] D. Z. Loesch , D. E. Godler , A. Evans , et al., “Evidence for the Toxicity of Bidirectional Transcripts and Mitochondrial Dysfunction in Blood Associated With Small CGG Expansions in the FMR1 Gene in Patients With Parkinsonism,” Genetics in Medicine 13, no. 5 (2011): 392–399. 21270637 10.1097/GIM.0b013e3182064362 PMC4022481

[45] E. Napoli , C. Ross‐Inta , S. Wong , et al., “Altered Zinc Transport Disrupts Mitochondrial Protein Processing/Import in Fragile X‐Associated Tremor/Ataxia Syndrome,” Human Molecular Genetics 20, no. 15 (2011): 3079–3092. 21558427 10.1093/hmg/ddr211 PMC3131047

[46] C. Giulivi , E. Napoli , F. Tassone , J. Halmai , and R. Hagerman , “Plasma Metabolic Profile Delineates Roles for Neurodegeneration, Pro‐Inflammatory Damage and Mitochondrial Dysfunction in the FMR1 Premutation,” Biochemical Journal 473, no. 21 (2016): 3871–3888. 27555610 10.1042/BCJ20160585 PMC7014977

[47] E. Napoli , G. Song , S. Wong , R. Hagerman , and C. Giulivi , “Altered Bioenergetics in Primary Dermal Fibroblasts From Adult Carriers of the FMR1 Premutation Before the Onset of the Neurodegenerative Disease Fragile X‐Associated Tremor/Ataxia Syndrome,” Cerebellum 15, no. 5 (2016): 552–564. 27089882 10.1007/s12311-016-0779-8 PMC5014718

[48] G. Pagano , A. Lyakhovich , F. V. Pallardó , L. Tiano , A. Zatterale , and M. Trifuoggi , “Mitochondrial Dysfunction in Fragile X Syndrome and Fragile X‐Associated Tremor/Ataxia Syndrome: Prospect Use of Antioxidants and Mitochondrial Nutrients,” Molecular Biology Reports 51, no. 1 (2024): 480. 38578387 10.1007/s11033-024-09415-7 PMC10997711

[49] A. Seritan , J. Cogswell , and J. Grigsby , “Cognitive Dysfunction in FMR1 Premutation Carriers,” Current Psychiatry Reviews 9, no. 1 (2013): 78–84. 25620901 10.2174/157340013805289635 PMC4304642

[50] D. Hessl , K. Mandujano Rojas , E. Ferrer , et al., “FMR1 Carriers Report Executive Function Changes Prior to Fragile X‐Associated Tremor/Ataxia Syndrome: A Longitudinal Study,” Movement Disorders 39, no. 3 (2024): 519–525. 38124331 10.1002/mds.29695 PMC11268876

[51] Y. H. Hwang , B. E. Hayward , M. Zafarullah , et al., “Both Cis and Trans‐Acting Genetic Factors Drive Somatic Instability in Female Carriers of the FMR1 Premutation,” Scientific Reports 12, no. 1 (2022): 10419. 35729184 10.1038/s41598-022-14183-0 PMC9213438

