# Supplementary figures and images for: Relationship Between Neurologic Symptoms and Signs and FMR1 Genotype in Premutation Carriers
Source: Ann Clin Transl Neurol. 2026 Mar 31:10.1002/acn3.70375. Online ahead of print. doi: 10.1002/acn3.70375 (PMC13394192; doi:10.1002/acn3.70375)

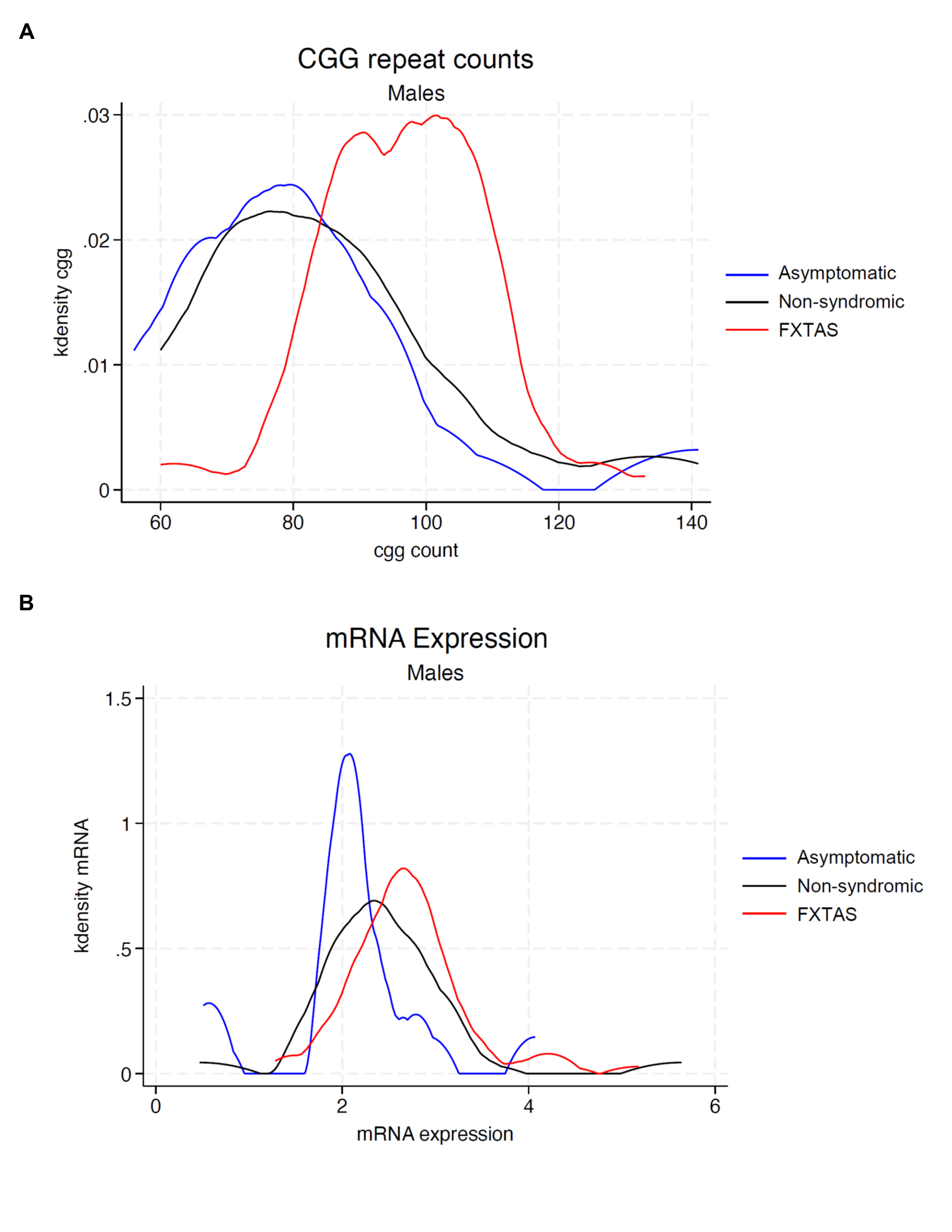

Supplement: Supplementary file 1 — Data S1: Kernel density estimate of CGG repeat number (a) and mRNA expression levels (b) in each of the three clinical categories for the male sample. [file ACN3-9999-0-s002.tif]

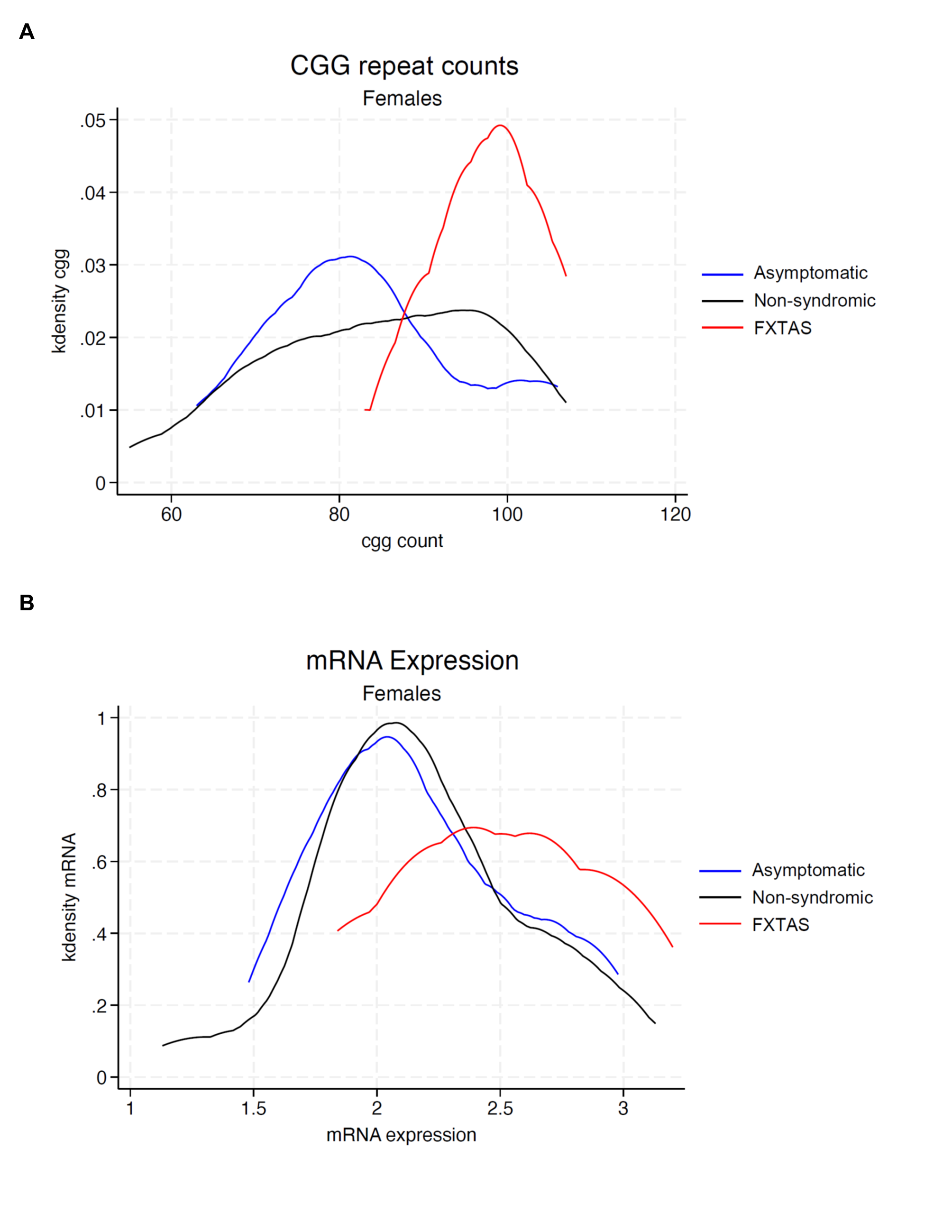

Supplement: Supplementary file 2 — Data S2: Kernel density estimate of mRNA expression levels (a) and CGG repeat number (b) in each of the three clinical categories for the female sample. [file ACN3-9999-0-s001.tif]
